# Cooperative update of beliefs and state-transition functions in human reinforcement learning

**DOI:** 10.1038/s41598-019-53600-9

**Published:** 2019-11-27

**Authors:** Hiroshi Higashi, Tetsuto Minami, Shigeki Nakauchi

**Affiliations:** 1 Graduate School of Informatics, Kyoto University, Kyoto, Japan; 2 Electronics-Inspired Interdisciplinary Research Institute, Toyohashi University of Technology, Toyohashi, Japan; 3 Department of Computer Science and Engineering, Toyohashi University of Technology, Toyohashi, Japan

**Keywords:** Decision, Learning algorithms, Human behaviour

## Abstract

It is widely known that reinforcement learning systems in the brain contribute to learning via interactions with the environment. These systems are capable of solving multidimensional problems, in which some dimensions are relevant to a reward, while others are not. To solve these problems, computational models use Bayesian learning, a strategy supported by behavioral and neural evidence in human. Bayesian learning takes into account beliefs, which represent a learner’s confidence in a particular dimension being relevant to the reward. Beliefs are given as a posterior probability of the state-transition (reward) function that maps the optimal actions to the states in each dimension. However, when it comes to implementing this learning strategy, the order in which beliefs and state-transition functions update remains unclear. The present study investigates this update order using a trial-by-trial analysis of human behavior and electroencephalography signals during a task in which learners have to identify the reward-relevant dimension. Our behavioral and neural results reveal a cooperative update—within 300 ms after the outcome feedback, the state-transition functions are updated, followed by the beliefs for each dimension.

## Introduction

To make correct choices, we need to predict future events based on past experiences. This is accomplished by learning to map between stimuli, actions, and outcomes. However, not all sensory inputs of observable objects are suitable as stimuli for the mappings that guide the decision-making process. In the real world, we face multidimensional problems in which only a few observable objects are relevant to the performance of a given task. If you want to safely cross the street, you will consider how far and how fast cars are, but ignore their colors and shapes^1^. Humans and animals select relevant dimensions based on past experiences. More generally, dimension identification improves performance and simplifies the decision-making process.

The reinforcement learning (RL) framework has successfully explained animal and human behavior in simple trial-and-error learning. However, little is known about the brain functions responsible for solving more complex problems, including multidimensional environments. Badre, Frank, and colleagues^2–4^ tackled a multidimensional problem by computational modeling of human behavior and functional magnetic resonance imaging (fMRI). In their experiment, cues with different shapes and orientations were used as stimuli; learners were presented with one cue and obtained a reward if they responded with the correct action. Only the shape or orientation of the cue was relevant to the correct cue-to-action mappings. More precisely, if the shape is the reward-relevant dimension, learners can respond with the correct action based solely on the shape without observing the orientation. This type of problem, which requires a learner to identify the reward-relevant dimension, will be referred to as a *dimension identification* problem throughout the paper. Problems that include dimension identification—such as hierarchical rules^2,5,6^, dimension attention^1,7,8^, multicue environment^9^, causal structure learning^10^, and informative cues^11^—have been tackled in computational neuroscience research.

Computational modeling is unraveling the brain activity connected with dimension identification^4,12^. A modeling study based on Bayesian learning suggests a learner has an internal model including *beliefs* that represent how much the learner believes that a given dimension is relevant to rewards^13–15^. These beliefs are also called reliabilities^4^, attention weights/biases^8^, and credit^9^. In addition to beliefs, the learner holds state-transition (or reward) functions that map the optimal actions to the current state in each dimension. Beliefs have a role in integrating the multiple state-transitions functions for all dimensions to determine the learner’s action^4,12^. When the learner observes a new experience, the internal model accounting for beliefs and state-transition functions is updated. The update can be implemented computationally in a Bayesian manner^4,12,16^. Neuroimaging research has revealed that this update process is associated with activities in the medial frontal cortex^9^, posterior parietal cortex, lateral prefrontal cortex, frontal pole^10^, and rostral premotor cortex^4^.

**Figure 1:**
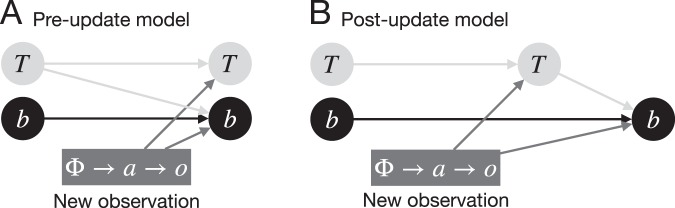
The two options of Bayesian learning for beliefs with state-transition functions. The symbols $t$, $b$, $\Phi$, $a$, and $o$ represent the state-transition function, beliefs, states, actions, and outcome, respectively, in our computational model (see *Learning model with beliefs* in the *Method* section). In (**A**) the pre-update model, the beliefs are updated with the state-transition functions that are not updated. In (**B**) the post-update model, the beliefs are updated with the state-transition functions that are already updated by the new experience.

However, their models accounting for beliefs were not enough to implement the learning process computationally. If this computation model using the Bayes rule is correct, the brain cooperatively updates the beliefs and the state-transition functions when the learner acquires an experience. For the implementation of the update, there are two options: (1) in *the pre-update model*, the beliefs are updated with the state-transition functions that are not updated; and (2) in *the post-update model*, the beliefs are updated with state-transition functions that are already updated by the new experience. Figure 1 depicts these two options. Previous studies introducing the concept of beliefs^2,4,10,12^ have discussed the structure of the internal model, but have failed to thoroughly describe how beliefs and state-transition functions cooperatively update. The post-update model was implicitly adopted. However, the order in which beliefs and state-transition functions update has not yet been investigated and is key to understanding how humans implement Bayesian learning.

This study aims to identify which of the two updating processes—the beliefs or the state-transition functions—comes first, by computational modeling, electroencephalography (EEG), and decoding. We designed a task in which learners must identify a reward-relevant dimension when presented with two dimensions. We confirmed that our computational models based on beliefs and state-transition functions could solve the task using simulated virtual learners. Next, we compared the computational models of the two options in terms of the accuracy of their fit to human behavior. Finally, neural signatures of the computational models were investigated by a trial-by-trial analysis of the outcome-related EEG signals. Thanks to the excellent temporal resolution of EEG, we could reveal the time dynamics of the cooperative update^17–21^. The neural signatures provide evidence that the brain either individually updates the beliefs and state-transition functions or cooperatively updates them together.

## Results

**Figure 2:**
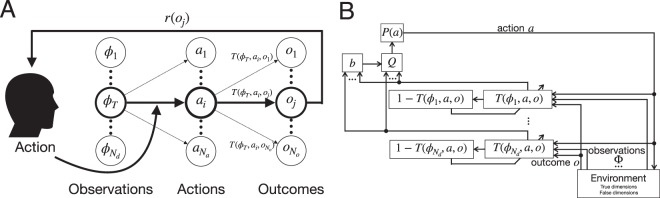
A problem with dimension identification. (**A**) Problem. A learner gives an action, and the outcome is provided according to the state-transition function for the reward-relevant dimension ${\varphi }_{T}$. (**B**) A learning model. The state-transition function $T({\varphi }_{n},a,o)$ is updated in parallel for each dimension. The corresponding belief for each dimension is computed as the posterior probability distribution of the observation, action, and outcome. The beliefs integrate the state-transition functions, and the learner determines an action based on the integrated one.

To investigate the brain process during dimension identification, we formulated a problem and computational learning models to solve it (Fig. 2, *Problem formulation* in the *Methods* section). Our models take into account beliefs and state-transition functions for each dimension. We considered that beliefs update based on the state-transition functions, with two possibilities for the order of update, according to the pre- and post-update models previously described in Fig. 1. In addition to these models, which make use of beliefs for dimension identification, we also tested conventional models, *the single model* and *the compound model* (see *Single and compound models* in the *Methods* section for details). The single and compound models do not take into account beliefs, but only the state-transition functions.

**Figure 3:**
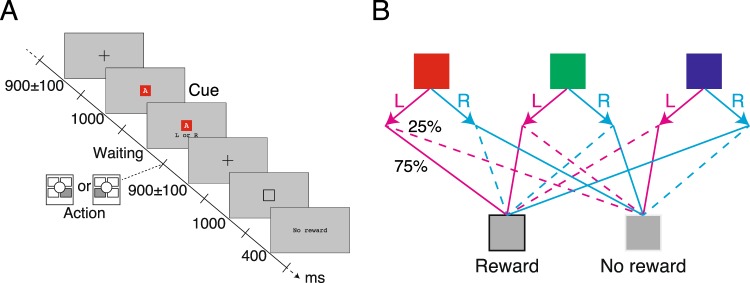
Experiment protocol. (**A**) Procedure followed by participants. A colored square with a letter of the alphabet was presented to the participants. The participants took an action with a left- or right-click of a trackball. About 900 ms after the response, the outcome was indicated by a white- or black-framed gray square. At the end of the trial, the outcome was confirmed to the participants by the phrase “Reward” or “No reward”. (**B**) An example of cue-action-outcome mapping, where color is the reward-relevant dimension. The solid lines connecting an action to a reward represent transitions of high probability (75).

Based on the formulated problem, we designed a cue-action mapping task where a learner identified the reward-relevant dimension when given two choices (Fig. 3). For a single trial in this task, a colored square with a letter in its center was presented to the learner as a cue. This cue had two dimensions (color and letter) and three states in each dimension (three different colors and letters). The learner had to identify the relevant dimension and find the optimal action for the corresponding state. Here, we present the results of simulated virtual learners and 29 human participants.

### Simulation with virtual learners

**Figure 4:**
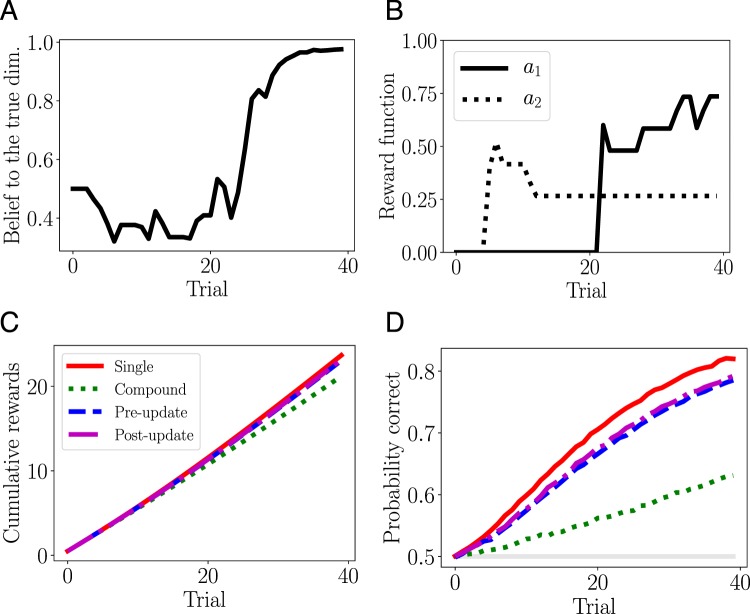
Simulations using virtual learners. (**A**) A typical evolution of the belief ${b}_{n}$ as a function of trials, for a post-update model learner. (**B**) Characteristic state-transition functions $Q({s}_{1,T},a)$ for the optimal (${a}_{1}$) and other (${a}_{2}$) in a post-update model learner. (**C**) The average cumulative rewards for each learning model. (**D**) The probability of selecting the correct (optimal) actions for each learning model. The gray line shows the chance level.

Through simulation with virtual learners, we confirmed that the computational models could solve our task. Figure 4A,B show the result of a simulation in which the learning rate $\alpha$ and the inverse temperature parameter $\tau$ were fixed at 0.2 and 5, respectively. As the learner accumulates trials, the belief that the reward-relevant dimension is ${d}_{1}$ converges to a value close to 1.0 (Fig. 4A). In Fig. 4B, the expected reward for the optimal actions (${a}_{1}$ for ${s}_{1,T}$) is higher than for the other actions. These results indicate that the virtual learner found the reward-relevant dimension and the correct mappings. The cumulative reward averaged over 100,000 blocks and the probability that the learners selected the correct actions are shown in Fig. 4C,D, respectively. The averaged rewards over blocks were $23.67\pm 4.37$, $21.20\pm 4.03$, $23.05\pm 3.79$, and $23.17\pm 3.40$ for the single, compound, pre-update, and post-update models, respectively. A one sample $t$-test showed that the cumulative reward for all models was greater than the chance level (20) of a random learner ($p < 0.001$). The cumulative reward curves suggest that the learners for all models successfully learned and selected the optimal actions. The probability of a correct response, which improved as the number of trials increased, corroborates this suggestion. This result shows that both the pre- and post-update models solved the task more efficiently than the compound model.

### Fitting to human behavior

**Figure 5:**
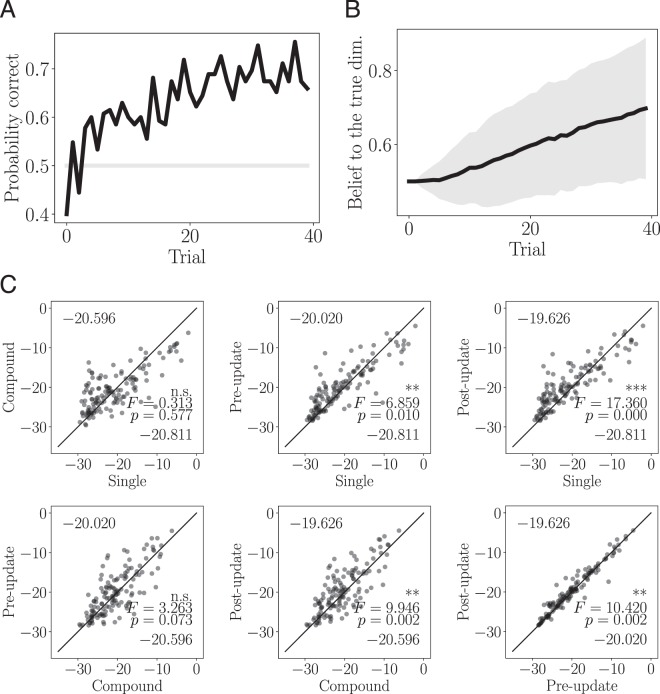
Fitting the models to human behavior. (**A**) Probability of selecting the correct (optimal) actions. The gray line shows the chance level. (**B**) Estimated belief in the reward-relevant dimension averaged over all blocks in the post-update model. The shaded regions represent the standard deviations. (**C**) Accuracy of fitting each model to human behavior, calculated using the log-likelihood. The plots show the accuracy for each pair of models. The numbers at the top and bottom of each plot are the means of the log-likelihoods of the models on the vertical and horizontal axis, respectively. The results of the ANOVA are shown on the right side of each plot.

We used the four computational models to predict human participants’ behavior during the experiment. Behavioral results from 26 participants were used for the analysis (data from three participants were excluded from the analysis due to problems with the EEG recording—for more details, see *EEG acquisition* in the *Methods* section). The average cumulative reward at the 40th trial is 22.76 $\pm$ 3.87. In Fig. 5A, we show for each trial the probability that the participants selected the optimal action. For the last (40th) trial, we performed a binomial test to compare the participants’ actions with those of an agent who chooses an action at random. It was found that the probability of selecting the correct action was significantly higher for the participants than for the random agent ($p=0.0002$). This significant result suggests that, in most of the blocks, the participants correctly identified the reward-relevant dimension and cue-action mapping.

Figure 5B shows the belief in the reward-relevant dimension, derived from fitting the behavior with the post-update model. Similarly to the simulated results shown in Fig. 4A, the belief increased as the number of trials increased. Figure 5C shows the fitting accuracies, which were evaluated by the log-likelihood for each learning model. A one-way repeated-measures analysis of variance (ANOVA) with the factor of the learning models, statistically significant differences were found in the single vs. post-update models, single vs. pre-update models, compound vs. post-update models, compound vs. pre-update models, and post-update vs. pre-update models, but not in the single vs. compound models and compound vs. pre-update models. In summary, the ANOVA results show that the post-update model produced the best prediction among the tested models. The average values of the optimized parameters were $$\{\eta ,\tau \}=\{0.431\pm 0.320,3.60\pm 5.18\},\{0.586\pm 0.319,5.65\pm 10.88\},$$ $$\{0.319\pm 0.276,4.45\pm 2.49\}\,,$$$$\{0.377\pm 0.295,4.82\pm 4.02\}$$ for the single, compound, pre-update, and post-update models, respectively.

### Event-related potentials

**Figure 6:**
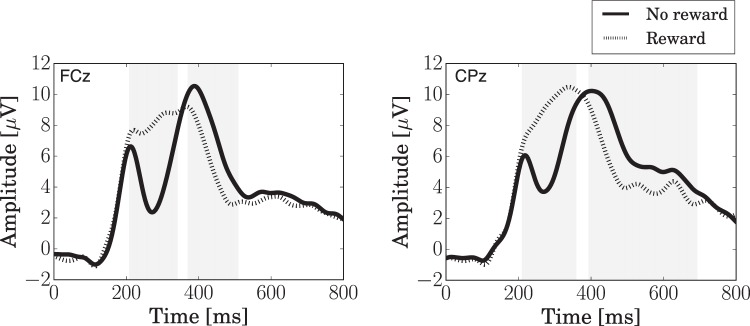
Feedback-related signals in channels FCz and CPz for rewarded vs. unrewarded trials. The shaded areas show time intervals in which there were significant differences ($p < 0.05$) in the potential between rewarded and unrewarded trials.

We analyzed the outcome-related potentials in the EEGs by a model-based analysis. We epoched the EEG signals such that the moment when the outcome was presented was set at 0 ms in the time window for the epoch (see *EEG acquisition* in the *Methods* section). Figure 6 shows the grand average of the outcome-related responses (rewarded vs. unrewarded trials). Differences in the EEG potentials between rewarded and unrewarded trials were calculated at specific moments in time and for each channel using a statistical $t$-test. We found significant differences between 250–350 ms for all presented electrodes, 400–500 ms for the frontal electrodes, and 400–600 ms for the parietal electrodes. We attribute the difference in potentials between 250–350 ms to feedback-related negativity (FRN)^22^, while the component observed beyond 400 ms was identified as P3^23^. FRN and P3 have been widely reported in RL studies^21^.

### Model-based analysis of EEG signals

To investigate the contributions of the computational models to the signals, we implemented a trial-by-trial approach to analyzing the EEG signals. We used regression to extract the effects of computational error signals (introduced by the models) in the EEG signal from various electrodes and at different points in time. Let us define the error for the state-transition as the *transition error* (${\delta }_{t}$), the error between an expected and an actual reward as the *reward error* (${\delta }_{r}$), and the discrepancy between prior and posterior updates in belief as the *belief error* (${\delta }_{b}$). The error signals were used as input for a generalized linear model (GLM)^24^, and the GLM predicted the EEG potentials. The prediction accuracy was evaluated by the deviance from the prediction. To find significant effects, we tested the accuracy with a likelihood-ratio test^25^; see *Model-based analysis of EEG signals* in the *Methods* section for procedural details.

**Figure 7:**
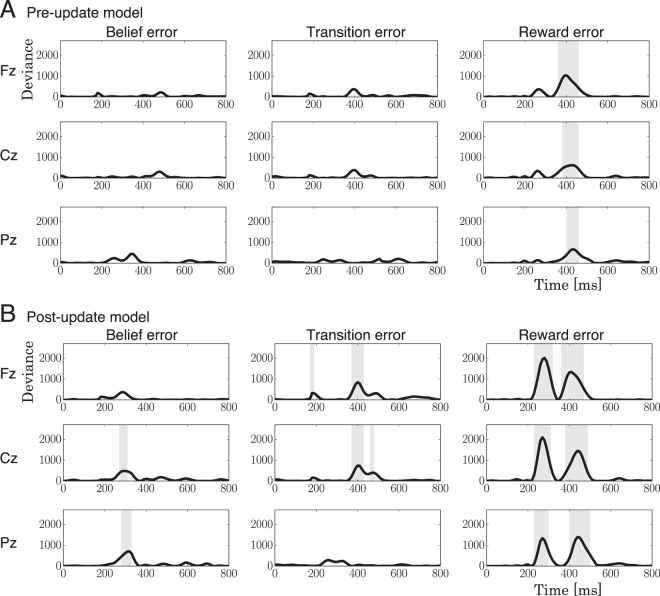
The deviances in the model-based analysis of EEG signals for (**A**) the pre-update model and (**B**) the post-update model. Shaded areas represent time intervals showing significant effects ($p < 0.05$) in the model-based analysis.

Figure 7 shows the prediction accuracy and the results of the statistical test. The effects of ${\delta }_{b}^{Po}$ are found within 280–340 ms in channels Cz and Pz. Through Fz and Pz, the effects of ${\delta }_{t}^{Po}$ are found within 370–420 ms, of ${\delta }_{r}^{\Pr }$ within 360–470 ms, and of ${\delta }_{r}^{Po}$ within 250–350 ms and 360–520 ms. By coinciding the latencies and spatial patterns, we concluded that the effects of the error signals on the EEG potential are caused by variations in the following event-related potentials (ERPs)^21,26^: the effect of the belief error in the post-update model (${\delta }_{b}^{Po}$) can be found in FRN, which is also affected by the reward error of the post-update model (${\delta }_{r}^{Po}$); last, the transition error of the post-update model (${\delta }_{t}^{Po}$) and the reward errors from both the pre-update and post-update models (${\delta }_{r}^{\Pr }$ and ${\delta }_{r}^{Po}$) had an effect on P3.

## Discussion

In this study, we tested a task in which a learner had to identify a reward-relevant dimension in a multidimensional environment through experiments with simulated virtual learners and human learners. We modeled a learning strategy for solving this task by introducing beliefs to each dimension. Simulations using virtual learners have shown that models that account for beliefs can solve the problem with dimension identification. Moreover, the post-update model in which beliefs update after the state-transition functions do predicts human behavior with higher accuracy compared with pre-update models. And last, EEG components reflecting error signals used to update the internal model were isolated.

Our study treats a specific step in the update mechanism for a problem with dimension identification. Previous studies^2,4,10,12^ proposed an internal model with beliefs and state-transition functions, supported by behavioral and neural evidence. In this internal model, when a learner acquires a new experience, beliefs and state-transition functions update simultaneously. However, previous studies did not consider the updating order and implicitly adopted the post-update model—i.e., beliefs are computed using state-transition functions that are already updated by the new experience. Because the post-update model exploits the latest experience to update all its internal elements, this model is more effective than the pre-update model. Indeed, we find that the post-update model fits well with behavioral and EEG data, suggesting that the brain updates state-transition functions and beliefs in this particular order.

The reward error defined in our study is fundamentally the same as the error signal that goes by the name of reward prediction error (RPE) in other RL literature^13,27–29^. In addition, the magnitudes of the RPE ($|{\rm{R}}{\rm{P}}{\rm{E}}|$) are the same as the transition error ${\delta }_{t}$, because the outcome was given as a binary value (reward/no reward or 0/1). In the model-based analysis, the fact that the FRN incorporates the reward error but not the transition error suggests a connection to both the valence and the magnitude of the RPE. This suggestion is supported by studies showing that the FRN reflects signed RPEs^18,30–34^ or is a temporally overlapped component of the valence and magnitude of the RPE^35,36^. However, our conclusion contradicts prior studies that claim the FRN is connected neither to the valence^28,37–42^ nor the magnitude^43–46^ of the RPE. It should be noted that the difference in the factors of the FRN variation could be caused by differences in experimental designs, such as outcome magnitudes and the existence of punishment^47^. Because our experimental design does not investigate these factors, we could not reach any conclusion as to which RPE components contribute to the FRN variation.

The FRN also reflected the belief error in the post-update model. As well as the results of the behavior fitting, this effect provides evidence that the brain updates beliefs with the updated state-transition functions. The belief error cannot be computed before the state-transition functions and beliefs are fully updated (see definition in (1)). This fact suggests that the update of the internal model is complete within 300 ms after the outcome. Nevertheless, despite our evidence that the update completes within 300 ms, we found an effect of the transition and reward errors on P3, which is also supported by Philiastides *et al*.^41^ and Bellebaum and Daum^40^, who reported that temporally overlapping but separate effects of the valence and magnitude of the RPE are found in P3. They suggested that the complete set of information that is to update the reward functions is available at the latency of P3, which is consistent with our observations. However, our evidence of the updating dynamics still contradicts their claim. If the update process completes within 300 ms, brain processes that affect the P3 are not considered to directly contribute to the process of updating. Because the reward error in the pre-update model also had an effect on the P3, it appears that multiple processes contribute to the P3 variation^21,48,49^, such as the reward magnitude^49^, the magnitude^26,50^ and valence^41,51^ of the RPE, the memory operation^20,23,52,53^, and adaptive mechanisms^46,51,54^.

This study has certain limitations. For our learning model, we did not consider the attention to each dimension. However, in our task, learners can adopt a learning strategy such that they pay attention to a specific dimension and update the state-transition function and belief only for this particular dimension^8,9^. This selective attention to specific dimensions improves the learning efficiency and is an important function called representation learning^1,7,15,55^. Although our task might not require dimensionality reduction^1^ by representation learning because of the small number of dimensions, representation learning models or attention-detective devices such as eye tracking systems^8,56^ would be needed to estimate the attention and accurately model human learning functions. Moreover, to apply our learning model to other problems, we should consider integrating model-free and model-based learning strategies^57^. Furthermore, a signal decomposition that can isolate overlapping components^58^ in EEG signals could be beneficial to reveal the details of the time dynamics in the brain. For example, Sambrook and Goslin^34^ successfully decomposed the overlapping components in feedback-related EEG signals in an RL problem by using principal component analysis.

In summary, this study provides insight into the time dynamics of brain processing in RL in a multidimensional environment. We found behavioral and neural evidence that humans solve this type of problem using a learning strategy in which the state-transition functions are updated, followed by the beliefs in each dimension. Moreover, our EEG measurements suggest that the update of the internal model is completed within 300 ms after a learner is provided with the outcome feedback. To our knowledge, this is a novel observation regarding the dynamics of the brain’s learning process.

## Methods

### Problem formulation

This section formulates a generalized problem for our task of dimension identification, after which a learning strategy to solve the problem is modeled. Computational error signals that are supposed to be computed while a learner is solving the problem are also defined.

#### Problem

Let us consider a problem in which a learner observes ${N}_{d}$ dimensions, ${\mathscr{D}}=\{{d}_{1},{d}_{2},\ldots ,{d}_{{N}_{d}}\}$. An element in ${\mathscr{D}}$ is the reward-relevant dimension denoted by ${d}_{T}\in {\mathscr{D}}$ that the learner does not know. The $n$th dimension has ${N}_{{s}_{n}}$ states, ${\mathscr{S}}_{n}=\{{s}_{n,1},{s}_{n,2},\ldots ,{s}_{n,{N}_{{s}_{n}}}\}$. At the beginning of each trial, the current states of all dimensions are presented to the learner. This combination of states is denoted by $\Phi ={\{{\varphi }_{n}\in {\mathscr{S}}_{n}\}}_{n=1}^{{N}_{d}}$. After observing the states, the learner selects an action out of $\mathscr{A}=\{{a}_{1},{a}_{2},\ldots ,{a}_{{N}_{a}}\}$. According to the action, the state transits to an outcome state out of $\mathscr{O}=\{{o}_{1},{o}_{2},\ldots ,{o}_{{N}_{o}}\}$. The probability of the transition to the outcome state is determined by the state in the reward-relevant dimension ${d}_{T}$, i.e., ${\varphi }_{T}$. Therefore, if the learner selects the action ${a}_{i}$, the state transits to an outcome state ${o}_{j}$ according to the transition probability $T({\varphi }_{T},{a}_{i},{o}_{j})$, as illustrated in Fig. 2A. At the end of the trial, an outcome state gives a reward defined by $r(o),o\in \mathscr{O}$. This problem thus includes both dimension identification and state-transition function learning.

#### Learning model with beliefs

We have modeled a learning strategy to solve the problem of a multidimensional environment. The learning strategy introduces a belief that probabilistically represents how much a learner believes a specific dimension. The idea behind the belief in this context is the same as in the the partially observable Markov decision process^59^. The learning model described below is illustrated as a block diagram in Fig. 2B.

The state-transition function $T(s,a,o)$ is updated at every trial. If the learner observes the transition to an outcome state $o$ from an observed state $\Phi$ by action $a$, the error signal is estimated as

$${\delta }_{n}=1-T({\varphi }_{n},a,o),$$

for all dimensions ($n=1,\ldots ,{N}_{d}$). Then, the state-transition function is updated by

$$T({\varphi }_{n},a,o)\leftarrow T({\varphi }_{n},a,o)+\eta {\delta }_{n}$$

and

$$T({\varphi }_{n},a,o^{\prime} )\leftarrow T({\varphi }_{n},a,o^{\prime} )(1-\eta ),$$

where $o^{\prime}$ represents the states in $\mathscr{O}$ except for $o$. The expected reward for the current state is also updated as

$$Q({\varphi }_{n},a)=\sum _{o\in \mathscr{O}}T({\varphi }_{n},a,o)r(o).$$

Belief is defined as the probability of whether or not a dimension is relevant to a reward. Let ${b}_{n}$ be the belief for dimension ${d}_{n}$. If the learner observes the transition to the outcome state $o$ by action $a$, the belief for dimension ${d}_{n}$ is given as a posterior probability^4,10,12,60^:

1 $$\begin{array}{lll}{b}_{n} & = & P({d}_{n}| \Phi ,a,o)\\  & = & \frac{P(o| {d}_{n},\Phi ,a)P({d}_{n}| \Phi ,a)}{P(o| \Phi ,a)}\\  & = & \frac{P(o| {\varphi }_{n},a)P({d}_{n})}{{\sum }_{m=1}^{{N}_{d}}P(o| {\varphi }_{m},a)P({d}_{m})}\\  & = & \frac{T({\varphi }_{n},a,o){b}_{n}^{^{\prime} }}{{\sum }_{m=1}^{{N}_{d}}T({\varphi }_{m},a,o){b}_{m}^{^{\prime} }},\end{array}$$

where $P(o| {\varphi }_{n},a)=T({\varphi }_{n},a,o)$, $P({d}_{n}| \Phi ,a)=P({d}_{n})$, and $b^{\prime}$ is the belief before updating.

The expected rewards for states and actions are given as

$$Q(\Phi ,a)=\mathop{\sum }\limits_{n=1}^{{N}_{d}}{b}_{n}Q({\varphi }_{n},a),$$

for $a\in \mathscr{A}$. The idea of computing the reward function for each action by weighting the action-reward functions with the beliefs has been proposed in a few previous studies^1,4,12^. The probability that the learner would take an action $a$ is given as

2 $$P(a)=\frac{\exp (\tau Q(\Phi ,a))}{{\sum }_{a^{\prime} \in {\mathscr{A}}}\exp (\tau Q(\Phi ,a^{\prime} ))},$$

where $\tau$ is the *inverse temperature parameter* controlling the extent to which the learner selects the higher-value action.

#### Error signals

After receiving the outcome feedback, the learner updates the state-transition functions and beliefs. In this update, signals that are supposed to be computed are defined as follows. The computational signal for a state transition (*transition error*) is defined as

$${\delta }_{t}=\mathop{\sum }\limits_{n=1}^{{N}_{d}}{b}_{n}(1-T({\varphi }_{n},a,o)).$$

This transition error comprises the unsigned reward prediction errors (RPEs)^13,27–29^ for all dimensions integrated by the beliefs. The signal for an expected reward (*reward error*) is defined as

$${\delta }_{r}=r(o)-Q(\Phi ,a).$$

This error comprises the signed RPEs integrated by the beliefs. The signal for a belief (*belief error*) is defined as

$${\delta }_{b}=\mathop{\sum }\limits_{n=1}^{{N}_{d}}{b}_{n}log\frac{{b}_{n}}{{b}_{n}^{^{\prime} }}={D}_{KL}(b\parallel b^{\prime} ).$$

This is a Kullback-Leibler (KL) divergence that represents the magnitude of the discrepancy from the prior ${b}_{n}^{^{\prime} }$ to the posterior ${b}_{n}$^10,11^, and ${D}_{KL}(\cdot \parallel \cdot )$ is an operator computing the divergence.

#### Update orders: pre- and post-update models

According to (1), the update needs the state-transition functions for all dimensions ${\{T({\varphi }_{m},a,o)\}}_{m=1}^{{N}_{d}}$. Here, we have a question: which updating process—beliefs or state-transition functions—comes first? That is, the order in which beliefs and state-transition functions are updated remains unclear. Our first hypothesis is that beliefs are updated with the pre-updating state-transition functions, which do not take into account the current outcome. We will refer to this formalism as the *pre-update model*. Our second hypothesis is that beliefs are updated with the post-updating state-transition functions, which are already updated according to the current outcome. The model which incorporates this order will be referred to as the *post-update model*. Figure 1 illustrates the update orders for these two models. Moreover, the error signals also depend on the order. The transition, reward, and belief errors are represented as $\{{\delta }_{b}^{\Pr },{\delta }_{t}^{\Pr },{\delta }_{r}^{\Pr }\}$ for the pre-update model and $\{{\delta }_{b}^{Po},{\delta }_{t}^{Po},{\delta }_{r}^{Po}\}$ for the post-update model.

#### Single and compound models

We compared the pre- and post-update models in terms of their performance in solving the problem using two conventional methods: in one dimension (the single model) and in multiple dimensions (the compound model). In the single model, we assumed that a learner observe only the reward-relevant dimension. Because no reward-irrelevant dimensions are taken into account, this model was used solely for reference. On the other hand, the compound model learned the state-action transition function for each compound state. In our experimental task, there were three states for each dimension—therefore, nine ($3\times 3$) compound states. This idea of the compound model is proposed as the *Flat expert*^4^ and the *Naïve RL*^1^.

### Experiment

#### Participants

29 individuals (25 male and 4 female) participated in the experiment. Their ages ranged from 21 to 25 years ($M=22.5$; $SD=1.2$). The participants had normal or corrected-to-normal visual acuity. All participants provided written informed consent. The experiment protocols were approved by the Committee for Human Research at the Toyohashi University of Technology, Aichi, Japan, and the experiment was conducted in accordance with the committee’s approved guidelines.

#### Experiment design

The participants were seated in front of an LCD display (VIEWPixx EEG, VPixx Technologies Inc.) on a chair in a dark, shielded room. Visual stimuli were sent using Psychtoolbox-3 and MATLAB R2011b (The MathWorks, Inc.).

During measurements, the participants attempted to achieve the highest cumulative reward by selecting actions. Figure 3A shows the procedure of our experimental task. In the center of the display, fixation cross (2.0$${}^{^\circ }$$$$\times $$ 2.0$${}^{^\circ }$$) was shown for 900 $$\pm $$100 ms, followed by the cue for the trial–a colored square (2.0$${}^{^\circ }$$$$\times $$ 2.0$${}^{^\circ }$$) with a letter at its center. The average luminance of a square was variable because, even though the luminance of a square was the same as the letter’s, the area occupied on the square varied with the letter. However, we believe that our results were unaffected because the areal difference was small and the letters were chosen randomly for each block. After 1,000 ms, the text “L or R” with a text size of $${2}^{^\circ }$$ was presented at $${5}^{^\circ }$$ below the center of the display. The cue square and the text remained on display until a response was given. Then, the participants selected “L” or “R” by a left- or right-button click of a four-button trackball, using their index fingers. After the response, the fixation cross was again presented for 900 $$\pm $$100 ms, followed by a gray square (2.0$${}^{^\circ }$$$$\times $$ 2.0$${}^{^\circ }$$) which told the participant whether a reward had been gained or not in that particular trial. After another 1,000 ms, the text “Reward” or “No reward” was shown for 400 ms to help the participant confirm the result of the reward. The participants repeated this trial 40–70 times in a block.

For each trial, the color of the cue square was randomly selected from blue, red, and green. For the letter of the cue square, a set of three letters for each block was randomly selected from the English alphabet. The letter for each trial was selected randomly from of the set. Therefore, the cue squares had nine combinations of colors and letters for each block. The gray square for indicating the reward to the participants had either a black or white frame to indicate “reward” and “no reward,” respectively. The correspondence between the frame brightness and the reward was counterbalanced across participants.

The reward for an action was delivered as follows. At the beginning of the experiment, the participants were told that one of the dimensions of the square (the color or letter) was relevant to the reward. However, The participants were not told which dimension would be relevant. Let $${d}_{T}$$ be the reward-relevant dimension which decided the optimal action and $${d}_{F}$$ be the reward-irrelevant dimension. Each dimension had three states, $$\{{s}_{1,T},{s}_{2,T},{s}_{3,T}\}$$ for $${d}_{T}$$ and $$\{{s}_{1,F},{s}_{2,F},{s}_{3,F}\}$$ for $${d}_{F}$$. The participants selected one of two options $$\{{a}_{1},{a}_{2}\}$$, which corresponded to either action “L” or action “R.” The correspondence between the action and the response was randomly determined for each block. The reward for each trial was probabilistically determined according to the current state of the reward-relevant dimension and the action. If the participant observed the state $${s}_{1,T}$$ and selected the action $${a}_{1}$$, the participant gained the reward at the probability of 75 This probabilistic rule can be represented by the conditional probability as $$P(r=1| {s}_{1,T},{a}_{1})=0.75$$, $$P(r=$$$$0| {s}_{1,T},{a}_{2})=0.25$$, $$P(r=1| {s}_{2,T},{a}_{1})=0.75$$, $$P(r=0| {s}_{2,T},{a}_{2})=0.25$$, $$P(r=1| {s}_{3,T},{a}_{1})=0.25$$, and $$P(r=0| {s}_{3,T},$$$${a}_{2})=0.75$$. On the other hand, for the reward-irrelevant dimension, the rules are $$P(r=0| {s}_{i,F},{a}_{j})=0.5$$ for $$i=1,2,3,\,j=1,2$$. In this setting, the optimal actions for $${s}_{1,T}$$ and $${s}_{2,T}$$ are $${a}_{1}$$, and $${a}_{2}$$ for $${s}_{3,T}$$. An example of the state-action-reward transition is shown in Fig. 3B.

The number of trials for each block depended on the participants’ actions. When the number of trials was over 40 and the participant selected the optimal action in at least 19 of the last 20 trials, the block ended. The block also ended when the participant performed 70 trials. Because all participants were paid the same, they were motivated to earn as many rewards as possible to finish early. If the block (or the whole experiment) ended early, participants would receive payment by shorter working time. Each participant performed more than six blocks; the first few were for practice and only the last five for our analysis.

The instructions to the participants are summarized as follows. The probability of gaining a reward depends on your response. If you respond with the optimal action, you have a 75 If you respond with the other action, there is only a 25 Each square has two properties, color and letter, and the optimal action depends on only one of them. The block ends if either the number of trials reaches 70 or if you respond with the optimal action in at least 19 of 20 consecutive trials. The optimal action and the reward-relevant dimension are changed for each block.

#### Simulation with virtual learners

To confirm that the learning model is able to solve our problem, we conducted a computer simulation. We used virtual learners that determined their actions according to the probability distribution defined by (2), with a learning rate $\alpha$ of 0.2 and an inverse temperature parameter $\tau$ of 5. To observe any trends in the independent virtual learners’ behavior, we ran 100,000 blocks with random cues. We observed trial-by-trial changes of the beliefs and state-transition functions of the learners.

#### Fitting to human behavior

The parameters in the single and compound models and the pre- and post-update models, $\eta$ and $\tau$, were fitted to human behavior data (participants’ actions). Because the performance in gaining the reward was different between blocks, even for a single participant, we grouped the data and performed the fitting by block, and not by participant. The fitting accuracy was evaluated using the likelihood of the actions, as formulated in (2). We found the parameters that achieved the maximum likelihood^61^ for each block. To find these parameters, the sequential least squares programming implemented in scipy as optimize.fmin_slsqp was used. We found the optimal values of $\eta$ and $\tau$ in the ranges $(0.001,0.999)$ and $(1,\inf )$, respectively. The initial values for the optimization were $0.1$ for $\eta$ and $5$ for $\tau$.

#### EEG acquisition

The EEG recording was performed at a sampling rate of 512 Hz with a 64-electrode cap, referenced to the averaged potential of both earlobes. The 64 active electrodes were positioned to cover the whole head according to the extended International 10/10 system. Additional signals were measured in extra active electrodes placed on the left and right earlobes, on the temple to the right side of the right eye, and on the left, upper, and lower sides of the left eye. A Butterworth bandpass filter (passband: 0.1–20 Hz, order: 4) was applied to the signals. Continuous EEG was epoched around the outcome onset (the time when the white- or black-framed square was presented from $-100$ to $1,000$ ms). An epoch for each trial was corrected using the $-100$ ms to 0 ms period as the baseline. The epochs in which the EEG and the vertical/horizontal electroculograms were larger than $\pm$80 $\mu$V were removed. The blocks with fewer than 10 epochs were also excluded, resulting in a total of 36 excluded blocks. For this reason, the blocks for three male participants were excluded in their entirety. The EEG epochs for 2,596 trials were left in total.

#### Model-based analysis of EEG signals

To find the event-related components in the EEG signals that significantly correlated to the trial-by-trial error signals in the pre- and post-update models, we used a multiple regression analysis of EEG signals with a GLM^24^. The RL error signals in the pre- and post-update models were used as the explanatory variables, and the EEG signal at certain electrodes and time points were used as the response variable. Two parameters—the learning rate $\eta$ and the inverse temperature $\tau$—for both the pre- and post-update models were obtained from fitting to the participants’ behavior in each model. In the GLM, we assumed that the response variable was generated using a Gaussian distribution with a linear link function. The EEG potential was calculated by averaging the epoch signals over a temporal window of $\pm$50 ms around every 10 ms from 0 to 800 ms from the onset of the outcome feedback. We tested all six error signals (${\delta }_{b}^{\Pr }$, ${\delta }_{t}^{\Pr }$, ${\delta }_{r}^{\Pr }$, ${\delta }_{b}^{Po}$, ${\delta }_{t}^{Po}$, and ${\delta }_{r}^{Po}$) as explanatory variables. Additionally, these error signals can be highly correlated with one another. Therefore, the deviance of an error signal was computed as the increase in the deviance of the model when accounting for all six error signals compared with accounting for only five error signals. In this way, correlation effects among error signals can be eliminated from the results. For instance, the fitting accuracy for ${\delta }_{b}^{\Pr }$ was derived as the increase in the deviance of the model when all errors were considered, compared with ${\delta }_{t}^{\Pr }$, ${\delta }_{r}^{\Pr }$, ${\delta }_{b}^{Po}$, ${\delta }_{t}^{Po}$, and ${\delta }_{r}^{Po}$. The fitting accuracy was statistically tested by a likelihood-ratio test^25^ implemented by a parametric bootstrap method^62^ (the number of sampling was 10,000).
